# Burden and spectrum of bacterial infections among sickle cell disease children living in Cameroon

**DOI:** 10.1186/s12879-017-2317-9

**Published:** 2017-03-15

**Authors:** Anastasie Nicole Alima Yanda, Jobert Richie N. Nansseu, Hubert Désiré Mbassi Awa, Sandra A. Tatah, Judith Seungue, Charlotte Eposse, Paul Olivier N. Koki

**Affiliations:** 1 Mother and Child Centre of the Chantal Biya Foundation, Yaoundé, Cameroon; 2 Faculty of Medicine and Biomedical Sciences, University of Yaoundé I, PO Box 1364, Yaoundé, Cameroon; 3 Douala Laquintinie Hospital, Douala, Cameroon

**Keywords:** Sickle cell disease, Children, Bacterial infection, Cameroon

## Abstract

**Background:**

Although sickle cell disease (SCD) children are highly susceptible to bacterial infections (BIs), there is a dreadful lack of data related to the burden and spectrum of BIs in sub-Saharan Africa (SSA), the highest affected region with SCD. This study aimed to determine the burden and spectrum of BIs among SCD children hospitalized in a pediatric reference hospital in Cameroon, a SSA country.

**Methods:**

We conducted a retrospective analysis of records of children hospitalized from November 2012 to August 2015 in the SCD unit of the Mother and Child Centre of the Chantal Biya Foundation, Cameroon. We enrolled all known SCD children aged 15 years or less, hospitalized for a suspicion of BI and who presented a positive culture of a body specimen.

**Results:**

A total of 987 SCD children were hospitalized during the study period. Cultures were positive for 96 patients (9.7%) among whom 60.4% males. Ages ranged from 6 to 192 months with a median of 53 (Interquartile range (IQR) 21–101) months. For children no more covered by the Expanded Programme on Immunization, only 13 (18.8%) had received the Pneumo 23® and Meningo A&C® antigens, and 12 (17.4%), the Typhim vi® and the *Haemophilus influenzae* type b antigens; 58 children (84.1%) had received no vaccine. The specimen yielding positive cultures were: blood (70.7%), urine (13.1%), pus (9.1%), synovial fluid (4.1%), cerebrospinal fluid (2.0%), and bone fragment (1.0%). The different types of infection included: urinary tract infections (13.5%), myositis (8.3%), arthritis (6.3%), osteomyelitis (4.2%), and meningitis (2.1%); the site of infection was unidentified in 65.6% of cases. The main bacteria included: *Salmonella sp.* (28.1%), *Staphylococcus sp.* (18.8%), *Klebsiella pneumoniae* (17.7%), *Escherichia coli* (10.4%), *Enterobacter sp.* (5.2%), *Acinetobacter sp.* (4.2%), *Streptococcus sp.* (4.2%) and *Serratia sp.* (4.2%).

**Conclusion:**

This retrospective analysis revealed 9.7% cases of BIs, mainly caused by *Salmonella sp.* (28.1%), *Staphylococcus sp.* (18.8%), *Klebsiella pneumoniae* (17.7%), and *Escherichia coli* (10.4%).

## Background

Sickle Cell disease (SCD) also referred to as Sickle Cell Anemia, is the most widespread and severe monogenetic disorder in the world, specifically in Africa south of the Sahara where it constitutes a real public health issue with 1–4% of babies born affected [1–3]. In Cameroon, around 20% of the population carries the sickle cell trait [4], and the disease prevalence is estimated at 0.6% in the general population. It is estimated that almost 4000 babies will be born with a major sickle cell syndrome each year (unpublished data).

SCD is particularly disastrous, mainly due to its acute and chronic complications, including painful vaso-occlusive events, cerebral vasculopathy, priapism, chronic kidney disease, acute chest syndrome, pulmonary hypertension and bacterial infections (BIs) among others [5–7]. Indeed, BIs are one of the prevailing complications of SCD and can occur either as an acute or a chronic condition. As such, they constitute a major cause of morbidity and mortality among SCD children and adults [8, 9]. A 20-years prospective study showed for instance that BIs led to 20–50% of deaths among SCD patients [8]. The increased susceptibility of SCD patients to BIs is multifactorial, including: (i) a functional asplenia; (ii) a default in complement activation; (iii) micronutrient deficiencies; (iv) a genetic predisposition, and (v) mechanical risk factors [8].

The organisms most commonly isolated from children with SCD are: *Streptococcus pneumoniae*, non-typhi *Salmonella sp.*, *Haemophilus influenzae* type b, *Escherichia coli*, *Acinetobacter sp.* and *Klebsiella sp.* [10, 11]. Evidence accumulated indicates a strong association between SCD and invasive bacterial disease among SCD Africans, especially greater for *Streptococcus pneumoniae*, non-typhi *Salmonella sp.* and *Haemophilus influenzae* type b [11, 12]. Moreover, it has been claimed a predominance of invasive pneumococcal infections below the age of five, and salmonella infections above 5 years old [13, 14].

While sub-Saharan Africa (SSA) is the highest affected region with SCD, there is a paradoxical dearth of data regarding the spectrum of BIs among SCD patients to guide the proper and efficient management of these threats across the region, especially in Cameroon, a SSA country. Willing to fill this critical gap, the present study was undertaken, the aim of which was to assess the burden and spectrum of BIs which have occurred among SCD children hospitalized in a pediatric reference hospital in Cameroon.

## Methods

### Study design and setting

We conducted a retrospective analysis of records of children hospitalized in the SCD unit of the Mother and Child Centre of the Chantal Biya Foundation from November 2012 to August 2015. This Centre, which has been well described elsewhere [7], is a pediatric reference hospital receiving patients coming from all over the country and the rest of the Central African sub-region. Nearly 700 SCD patients are regularly followed-up, with an average of 400 hospitalizations per year. The body specimens were analyzed at the Centre Pasteur of Cameroon. Located in Yaoundé and at a 2-min walking distance from the hospital, the Centre Pasteur of Cameroon is the laboratory of reference in the country; it uses international standard procedures and goes along a regular and rigorous internal and external control of quality.

### Study participants

During the study period, we consecutively and exhaustively included all known SCD children aged 15 years or less, who have been hospitalized for a suspicion of BI and presented a positive result after culture of a body specimen (blood, urine, cerebrospinal fluid, bone fragment, synovial fluid or pus). Samples were collected in the unit and transported without delay to the Centre Pasteur of Cameroon where they were analyzed. Bacterial isolations were conducted using standard culture milieus such as Cystine Lactose Electrolyte Deficient, Chapman, Mac Conkey, chocolate (agar cooked/fresh blood), or polyvitex chocolate, depending on the type of specimen to be analyzed. Identification of the bacteria used different specific antibiotic galleries. Poly-microbial cultures were not considered in the present work, neither were contaminations (identified as positive cultures in patients with fever dropping without any antibiotherapy, or good remission with an antibiotherapy described, after the antibiogram, as resistant to the incriminated bacterium).

### Data collection

We used a preconceived and standardized questionnaire for data abstraction. Data were collected on age, sex, vaccination status after the Expanded Programme on Immunization (EPI) (especially regarding Pneumo 23®, Meningo A&C®, *Haemophilus influenzae* type b and Typhim vi® antigens), clinical signs and symptoms at presentation (fever, vaso-occlusive crises), site of infection and type of sampling performed, isolated bacterium, treatment and outcome in the course of hospitalization.

### Statistical methods

Data were coded and entered using Microsoft Excel 2010, and analyzed with SPSS software version 20.0 (IBM SPSS Inc., Chicago, Illinois, USA). Results are presented as frequency (percentage) for qualitative variables, and median (interquartile range (IQR)) for quantitative ones. Comparison between qualitative variables used the Chi-2 test, and results were considered statistically significant when *p* < 0.05.

### Ethical statement

Before commencing this study, authorizations were obtained from administrative authorities of the Mother and Child Centre, and an ethical clearance was granted by its Ethical Review Board. All the procedures used in the present study were in keeping with the current revision of the Helsinki Declaration. As the study was retrospective, patients’ consents could not be obtained. However, the study was approved by the association of parents of SCD children of the Mother and Child Centre which includes the majority of parents of these diseased children formerly followed-up or hospitalized in the SCD unit.

## Results

On the whole, 987 children suffering from SCD were hospitalized during the study period; cultures were positive for 96 patients (9.7%). Patients’ ages ranged from 6 to 192 months, with a median of 53 (IQR 21–101) months. There were 58 males (60.4%), hence a M/F sex ratio of 1.53/1. Twenty seven children (28.1%) were aged less than 24 months (Table 1). Among the 69 patients equal or above 24 months of age, only a few had received vaccine recalls after the EPI: 13 (18.8%) for the Pneumo 23® and Meningo A&C® antigens, and 12 (17.4%) for the Typhim vi® and the *Haemophilus influenzae* type b antigens; 58 children (84.1%) had received no vaccine (Table 1). Fever was noticed among 90 patients (93.5%), and vaso-occlusive crises were present among 80 children (83.3%; Table 1).

**Table 1:** Demographic characteristics, main symptoms and sites of infection

Characteristic	Number	Percentage (%)
Age groups (years)		
< 2	27	28.1
≥ 2	69	71.9
Sex		
Male	58	60.4
Female	38	39.6
Vaccination coverage		
EPI (0 to 12 months)	96	100.0
Overall coverage from 24 months (*N* = 69)	11	15.9
Pneumo 23 (*N* = 69)	13	18.8
Meningo A&C (*N* = 69)	13	18.8
Typhim vi (*N* = 69)	12	17.4
*Haemophilus influenzae* type b (*N* = 69)	12	17.4
Vaso-occlusive crises: yes/missing	80/07	83.3/7.3
Fever: yes/missing	90/00	93.8/0.0
Type of infection		
Myositis	08	8.3
Osteomyelitis	04	4.2
Arthritis	06	6.3
Urinary tract infection	13	13.5
Meningitis	02	2.1
Septicemia	63	65.6
Body specimen cultured^a^		
Blood	66	68.8
Blood + synovial fluid	01	1.0
Blood + bone fragment	01	1.0
Blood + pus	02	2.1
Pus	07	7.3
Cerebrospinal fluid	02	2.1
Urine	13	13.6
Synovial fluid	03	3.1

*EPI* Expanded Programme on Immunization; ^a^Total: 99 specimen sampled for culture

The specimen yielding positive cultures were: blood (70.7%), urine (13.1%), pus (9.1%), synovial fluid (4.1%), cerebrospinal fluid (2.0%), and bone fragment (1.0%). The different types of infection were: urinary tract infections (13.5%), myositis (8.3%), arthritis (6.3%), osteomyelitis (4.2%), and meningitis (2.1%). Besides, the site of infection was unidentified (bacterial septicemia) in 63 patients (65.6%; Table 1). There was no difference between males and females (*p* = 0.779), and between the under-5 and 5+ years old (*p* = 0.291) with regard to the type of infection.

Bacteria were Gram negative in 74 cases (77.1%), with no difference between males and females (*p* = 0.396), and the under-5 and 5+ years (*p* = 0.221). Table 2 is representative of the different bacteria recorded with regard to the type of infection presented. The main bacteria included: *Salmonella sp.* (28.1%), *Staphylococcus sp.* (18.8%), *Klebsiella pneumoniae* (17.7%), *Escherichia coli* (10.4%), *Enterobacter sp.* (5.2%), *Acinetobacter sp.* (4.2%), *Streptococcus sp*. (4.2%), and *Serratia sp.* (4.2%). The majority of cases of septicemias were caused by *Staphylococcus sp.* (24.6%), *Salmonella sp.* (24.6%) and *Klebsiella pneumoniae* (16.9%). *Escherichia coli* was responsible for the majority of cases of urinary tract infections (53.8%) and *Salmonella sp.*, for all cases of myositis, The two cases of meningitis were caused by *Streptococcus pneumoniae* (Table 2). Moreover, five patients presented an infectious recurrence; the most common bacterium in cause was *Staphylococcus sp.*, and the site of infection was not identified in the majority of cases (Table 3).

**Table 2:** Different germs recorded considering the type of infection

Germ	Type of infection						**Total**
	Myositis	Osteomyelitis	Arthritis	Urinary tract infection	Meningitis	Septicemia	
*Acinetobacter baumanii*						3	03
*Acinetobacter (others)*						1	01
*Alloiococcus otitidis*						1	01
*Citrobacter koserii*						1	01
*Corynebacterium sp.*						1	01
*Enterobacter aerogenes*						1	01
*Enterobacter cloacae*			1			3	04
*Enterococcus sp*						1	01
*Escherichia coli*				7		3	10
*Klebsiella pneumoniae*			2	4		11	17
*Proteus mirabilis*				1			01
*Proteus vulgaris*						1	01
*Pseudomonas fluorescens*				1			01
*Salmonella dublin*		1				1	02
*Salmonella enterica serovar stanleyville*			1			1	02
*Salmonella enteritidis*	2					3	05
*Salmonella sp. (others)*	5	1	1			9	16
*Salmonella typhi*	1		1				02
*Serratia liquefaciens*		1					01
*Serratia marcescens*						3	03
*Staphylococcus aureus*		1				1	02
*Staphylococcus epidermidis*						4	04
*Staphylococcus haemolyticus*						4	04
*Staphylococcus hominis*						6	06
*Staphylococcus sciuri*						1	01
*Staphylococcus (others)*						1	01
*Streptococcus pneumoniae*					2		02
*Streptococcus of group C*						1	01
*Streptococcus (others)*						1	01

**Table 3:** Cases of infectious recurrence

Patient	First episode			Second episode		
	Age (months)	Germ	Type of infection	Age (months)	Germ	Type of infection
1	11	*Staphylococcus sciuri*	Septicemia	21	*Staphylococcus haemolyticus*	Septicemia
2	52	*Salmonella sp*	Septicemia	75	*Staphylococcus hominis*	Septicemia
3	123	*Staphylococcus sp*	Septicemia	133	*Enterobacter cloaccae*	Septicemia
4	159	*Enterobacter cloaccae*	Arthritis	170	*Corynebacterium sp*	Septicemia
5	17	*Escherichia coli*	Urinary tract infection	28	*Escherichia coli*	Urinary tract infection

Noteworthy, the antibiotherapy, which was started after the sampling had been done and before waiting for bacteriological results, comprised: as first line ceftriaxone and gentamycin, and as second line ofloxacin/ciprofloxacin and amikacin (or licomycin, depending on the results of the antibiogram as soon as available). The outcome was favorable for 82 patients (85.4%), but worse for the other ones among whom 4 patients ending up with osteo-articular sequelae (4.2%); 7 patients (7.3%) were transferred to undergo surgery (for osteomyelitis or myositis), and 3 patients (3.1%) died. Duration of hospitalization in the SCD unit varied between 1 and 21 days with a median of 9 (IQR 8–12) days.

## Discussion

Bacterial infections (BIs) are a major cause of morbidity and mortality among SCD children and adults [8], but there remains a crucial dearth of data regarding its burden in SSA countries such as Cameroon. We aimed to assess the burden and spectrum of BIs in our context. Results from the present retrospective analysis showed a 9.7% prevalence of BIs, comparable to the 6% obtained by Williams et al. [11]. On the contrary, our findings are lower that what were observed by Diop et al. [15] and Diakité et al. [16], respectively 50% and 40%. This low prevalence (9.7%) could be explained by the fact that due to lack of financial means, many patients’ parents were incapable of doing all the required exams. We noticed for instance that only 46.2% of the bacteriological tests required were performed. Furthermore and in the large majority of cases, only one blood culture (instead of at least three per patient) was performed, lowering therefore our probability of catching up the bacterium. Another point to be raised is that in Cameroon, newborns are not systematically screened for SCD. In this study we considered only known SCD patients, which could have underestimated the real burden of BIs in this vulnerable population.

In Cameroon indeed, newborn hemoglobinopathy testing is neither available nor implemented universally compared to developed countries. Only parents of SCD children who are being followed in specialized centers will be educated and counselled on infection prophylaxis with oral penicillin (to be paid by the parent), and to bring their children to the hospital when the temperature rises. Since 2011, the Prevenar 13® vaccine was introduced in the Cameroon Expanded Programme on Immunization, which is administered free of charge to all children at 6, 10 and 14 weeks of age. Additionally, a 23-valent pneumococcal vaccine as well as some others (Typhim vi, Meningo A&C) are proposed at 24 months to SCD children who are followed-up, but it is not given free of charge; hence only parents who can afford will vaccinate their kids.

This explains perhaps why we observed in this study, a low vaccination coverage after the EPI, around 15.9%. Similarly, previous African reports have bolstered a low vaccination coverage after the EPI in the young pediatric population, around 6.2–32.5% in the Gabonese public sector with pneumococcal antigen coverage equaling 0.8%, and around 10.8–13% in Cameroon [17, 18]. It is worth mentioning that our SCD children’s parents are always sensitized and educated on the importance of vaccinating their vulnerable kids and reminded in this regard, with regular campaigns being organized by the association of parents of SCD children. However, the costs of vaccines remain high for a large majority of parents, perhaps justifying the low coverages we have witnessed. Subsidization of vaccinations could thereby constitute a valuable solution to increase the vaccination coverage in this group of children. Nevertheless, studies willing to capture the factors explaining these low coverages are warranted.

Most of bacteria (71.4%) were identified through blood culture, blood being easily accessible to be sampled. Besides, a great number of infections including localized ones, have a bacteremic phase during which the bacterium can be picked up through culture of blood [15, 16]. The majority of bacteria identified (77.1%) were Gram negative, concurring with Diop et al.’s findings [15]. By contrast, Williams et al. observed a higher proportion of Gram positive bacteria (41%), *Streptococcus pneumoniae* especially [11]. Our observations could be the result of the introduction of the Prevenar 13® antigen in the Cameroon EPI since 2011, the antibio-prophylaxis (with oral penicillin) on which almost all children followed in the SCD unit and aged 2 to 5–6 years are placed, and the practice of self-medication with abusive and inappropriate intake of antibiotics. This access to non-prescribed antibiotics may have biased the different specimen culture results we obtained in this setting. It is therefore understandable why only 2 cases of *S. pneumoniae* infections were recorded; these two children who presented meningitis had neither received the Prevenar 13® nor the Pneumo 23® antigens.

The bacterial ecology was dominated by *Salmonella sp.* (28.1%), corroborating previous reports [13, 15, 16, 19]. This predominance is related to the digestive translocation of Salmonellas (ischemic embrittlement) favored by microvascular occlusions [8, 10, 15, 16]. Many infections were also caused by *Klebsiella pneumoniae* (17.7%), not often found in these proportions according to previous reports [11, 15, 16]. *K. pneumoniae* was the second bacterium responsible for urinary tract infections (30.8%), in line with results from Asinobi et al. (18.9%) and Mava et al. (24.6%) [12, 20]. *Escherichia coli* was responsible for 10.4% of infections, mainly urinary tract infections (53.8%) still concurring with the literature [12, 13, 20].

*Acinetobacter sp.* was the fourth bacterium reported by Williams et al., found in 7% of cases [11], hence almost comparable to the 4.2% we have recorded. *Staphylococcus sp.* was responsible for 18.8% of BIs, corroborating previous studies [13, 19, 21, 22]. These bacteria were mainly represented by those known to be commensals, hence a possibility of contaminations. In fact, due to lack of finances to perform more explorations, it was difficult to differentiate between real infections caused by these bacteria (identified through only one bacteriological exploration) and a simple contamination of the sample cultured. However, the concerned patients presented an inflammatory syndrome and got better only after proper and adapted antibiotherapy, confirming thereby the initial suspicion. The absence of *H. influenzae* infections in the present report may be explained by the introduction (in 2008) of three doses of the *Haemophilus influenzae* type b antigen in the Cameroon EPI, notably at 6, 10 and 14 weeks of age, and perhaps abusive use of antibiotics as self-medication.

We recorded 5 cases of infectious recurrence reflecting the high susceptibility of SCD patients to BIs, driven by a functional asplenia, a default in complement activation, micronutrient deficiencies, and mechanical and genetic factors [8]. Three patients died: one 2 years old boy who died at 24 h of hospitalization from meningitis caused by *Streptococcus pneumoniae*, and a 5 years old male child at 48 h from a severe sepsis caused by a non-typhi *Salmonella sp.* The third case was a female child of 7 years who was suffering from a severe sepsis with multifocal sites caused by a non-hospital acquired multi-resistant *Enterobacter cloacae*; she was transferred to the emergency care unit where she died after 3 months of hospitalization.

Unfortunately, this study presents a number of flaws. First, the retrospective analysis of this work precluded us from obtaining all the information necessary, and having a control group to investigate some factors driving the occurrence of BIs in our milieu. Second, parents’ financial constraints and lack of funding impeded the realization of more bacteriological explorations which could have permitted to depict and confirm more cases of BI; for the same reasons, only one blood culture (instead of at least three per patient) was performed for most patients, hence diminishing considerably our probability of catching up the causal agent. Moreover, a free access to non-prescribed antibiotics in our context may have constituted a source of bias towards our results. Third, our analyses are based only on known SCD children, in a context where newborns are not systematically screened for SCD; this could have led to an underestimation of the real burden of BIs. Fourth, SCD children were recruited from only one hospital, perhaps hindering the generalization of results obtained. Nonetheless, the study was conducted in a reference hospital with patients coming from all over the country and the Central African sub-region. Besides, the study period of almost 3 years permitted to record a great number of cases.

## Conclusion

This 3-years retrospective analysis permitted to record 96 cases (9.7%) of bacterial infections among SCD children living in Cameroon. The bacterial ecology was mainly represented by *Salmonella sp.* (28.1%), *Staphylococcus sp.* (18.8%), *Klebsiella pneumoniae* (17.7%), *Escherichia coli* (10.4%), *Enterobacter sp.* (5.2%), *Streptococcus sp.* (4.2%), *Acinetobacter sp.* (4.2%), and *Serratia sp.* (4.2%). Substantial efforts to vaccinate SCD children after 12 months of age (when they leave the EPI) and increase the vaccination coverage in this group of vulnerable children could help to reduce the burden of BIs in SCD children. Patient’s parents should be continuously sensitized and educated on the importance of vaccinating their kids, observe the penicillin antibio-prophylaxis and bring their kids to the hospital as soon as the temperature rises. Our clinicians must be adequately trained to manage these infections, bearing in mind that broad spectrum antibiotherapy should be started without waiting for bacteriological results. On the other hand, we believe it is high time our Governments think of subsidizing the management of SCD children for those parents who cannot pay. Cost-benefit studies are warranted in this regard.

## Abbreviations

BI: Bacterial infection
EPI: Expanded Programme on Immunization
IQR: Interquartile range
SCD: Sickle cell disease
SSA: Sub-Saharan Africa
